# Hybrid material of sustainable newspaper waste-derived cellulose for histamine sensing in kombucha tea

**DOI:** 10.1016/j.fochx.2025.102578

**Published:** 2025-05-26

**Authors:** Pongpat Sukhavattanakul, Nadnudda Rodthongkum, Sarute Ummartyotin

**Affiliations:** a Department of Materials and Textile Technology, Faculty of Science and Technology, Thammasat University, Pathumtani, Thailand; b Department of Chemistry, Faculty of Science, Chulalongkorn University, Bangkok 10330, Thailand; c Center of Excellence in Responsive Wearable Materials, Metallurgy and Materials Science, Research Institute, Chulalongkorn University, Soi Chula 12, Phayathai Road, Pathumwan, Bangkok 10330, Thailand; d Center of Excellence on Petrochemical and Materials Technology, Chulalongkorn University, Bangkok, Thailand

**Keywords:** Cellulose-based newspaper waste, Diamine oxidase, Histamine sensing, Hydroxypropyl methylcellulose, Kombucha

## Abstract

This study introduces a novel approach for sensing histamine by developing sensors from cellulose-based newspaper waste. The cellulose-based sensors, featuring diamine oxidase (DAO) as the sensing agent in cooperation with horseradish peroxidase (HRP) and 3,3′,5,5′-tetramethylbenzidine (TMB) solutions, are designed and assembled for targeted histamine detection. Experimental results demonstrate the sensor's exceptional sensitivity, with the observed color change serving as a reliable indicator of histamine concentration. In the presence of peroxidase and H_2_O_2_, the hydroxypropyl methylcellulose (HPMC)/DAO-immobilized enzyme-hydrolyzed cellulose-based film sensor gets oxidized, resulting in a blue spot. The highest sensitivity to histamine (3.30 mg/mL) was exhibited by the 3 *w*/w % HPMC/DAO-immobilized enzyme-hydrolyzed cellulose-based film, which showed the lowest b* value (−8.85 ± 0.01). This study showcases the potential of these films as an effective and cost-efficient platform for histamine detection in fermented beverages, offering promising applications in food safety screening. Ultimately, the research underscores the environmental sustainability of the HPMC-based hybrid material.

## Highlights

• Hydroxypropyl methylcellulose/diamine oxidase-immobilized film created from enzyme-hydrolyzed cellulose enhances sustainability.
• Utilizes newspaper waste to develop a cellulose-based film for effective histamine sensing.
• Visual detection enabled through a color transition in response to histamine.
• Diamine oxidase-immobilized enzyme-hydrolyzed cellulose showed superior quality compared to unloaded counterpart.

## Graphical abstract

**Figure:**
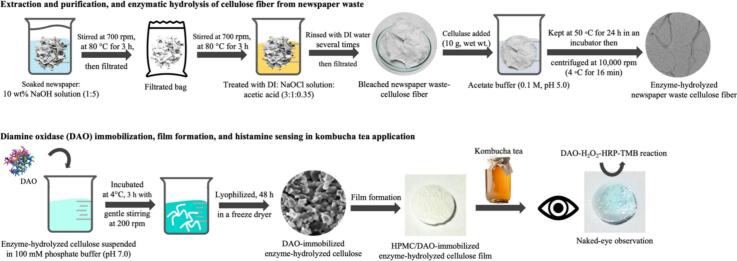

## 1 Introduction

The proposed research aims to develop a sustainable cellulose-based sensor for detecting histamine in kombucha, addressing an important consumer safety concern. Elevated histamine levels in fermented beverages can pose significant health risks, making effective monitoring essential for consumer safety and product quality. This study seeks to create a histamine sensor with enhanced detection capabilities specifically for kombucha. Current detection methods have limitations that this research intends to overcome by using a hybrid cellulose-based material sourced from renewable resources. This innovative sensor is designed to significantly improve histamine detection in kombucha.

Histamine, a biogenic amine linked to food safety, has gained significant attention in the realm of fermented beverages such as kombucha. This study is set against the backdrop of kombucha's rising popularity and the pressing need for effective histamine monitoring in liquid food products. The introduction of cellulose-derived histamine sensors, made from a hybrid material that combines polysaccharides with cellulose-based newspaper waste, represents a notable advancement in sustainable and efficient sensing solutions. The experiment focuses on testing the sensor's performance in kombucha, emphasizing its sensitivity, selectivity, and real-time monitoring capabilities. Results demonstrate the successful development of these cellulose-derived sensors for histamine detection in kombucha. The HPMC-based hybrid material effectively detects histamine with high sensitivity and selectivity. Notably, the color change observed in the film sensors—caused by the enzymatic reaction between DAO and histamine—serves as a reliable indicator of histamine concentration. DAO is an important enzyme that is becoming increasingly relevant in food safety analysis, particularly for detecting histamine in fermented beverages like kombucha tea (da Silva et al., 2002). As kombucha's popularity grows, ensuring its safety for consumption becomes increasingly important. Histamine, a biogenic amine that can accumulate during fermentation, poses health risks at high levels. DAO offers a promising approach for histamine detection due to its specificity and sensitivity (Biji et al., 2015). This enzyme catalyzes the oxidative deamination of histamine, producing hydrogen peroxide, which can be measured to quantify histamine levels. The resulting hydrogen peroxide can then be coupled with a chromogenic substrate, leading to a visible color change that serves as a clear indicator of histamine presence (Maintz & Novak, 2007). This color sensor appearance provides a user-friendly and rapid method for histamine detection, making it particularly valuable for on-site testing. Utilizing this DAO-based colorimetric assay for histamine analysis in kombucha tea offers a potential method for quality control and safety assurance in the rapidly expanding functional beverage market, combining enzymatic specificity with easy-to-interpret visual results. Recent advancements utilize hydrophobic deep eutectic solvents (HDESs) for the rapid preconcentration of Se (IV) from tea samples through a synergistic cloud point extraction method (HDES-RS-CPE) followed by hydride generation atomic absorption spectrometry (HG-AAS). A rapid synergistic cloud point extraction method utilizing hydrophobic deep eutectic solvents is employed to enhance the determination of selenium levels in tea samples. This approach is integrated with hydride generation atomic absorption spectrometry to achieve accurate results (Ridha et al., 2024). Additionally, the research explores the potential of MgO-based chitosan nanoparticles to enhance oxidant and dye scavenging properties, positioning them as promising candidates for antioxidant coatings and efficient photocatalysts (Ben Amor et al., 2023).

Several innovative approaches for histamine analysis have been developed over the last decade (Patange et al., 2005). Mopper and Sciacchitano (Mopper & Sciacchitano, 1994) described the use of capillary zone electrophoresis to determine histamine in fish, with UV detection at 210 nm. Other techniques used for determining histamine in fish include the use of an oxygen sensor-based assay using purified amine oxidase (et al., 1994), a solid phase assay based on the coupling of DAO to a peroxidase/dye system, monoclonal antibody-based ELISA (Serrar et al., 1995), a DAO-based amperometric biosensor for total histamine, putrescine, and cadaverine, and the use of an electrochemical biosensor for biogenic amine contents of foods (et al., 1996). The discussion delves into the sensor's ability to detect histamine in kombucha, emphasizing its advantages in terms of sensitivity and selectivity. Comparative analyses with existing technologies underscore the innovative contributions of the developed sensor in liquid food product applications. The study concludes by highlighting the environmental sustainability of the HPMC-based hybrid material and its crucial role in ensuring food safety through accurate histamine detection in kombucha. This research not only advances histamine sensing technology but also underscores the potential of HPMC as a biocompatible and effective component in cellulose-derived histamine sensors for liquid food products. This study presents a comprehensive exploration of cellulose-derived histamine sensors tailored for histamine sensing in kombucha, leveraging a hybrid material derived from polysaccharide and cellulose-based newspaper waste. The integration of sustainable materials not only addresses environmental concerns but also enhances the sensor's sensitivity and selectivity for histamine detection in this specific liquid fermented beverage. A thorough characterization process ensures the hybrid material's suitability for histamine sensing in kombucha, offering a sustainable solution for monitoring histamine levels in liquid environments.

The utilization of newspaper waste in our enzyme-hydrolyzed cellulose-based film exemplifies circular economy principles by promoting resource efficiency and waste reduction. By repurposing material that would otherwise contribute to landfill waste, we not only minimize environmental impact but also support sustainable practices. This approach highlights the potential for transforming waste into valuable products, thereby creating economic opportunities while addressing pressing environmental challenges. Such innovations are vital for advancing a more sustainable materials cycle and reducing reliance on virgin resources (Elroi et al., 2023). This alignment with circular economy principles illustrates how industries can shift towards more sustainable practices, reducing their ecological footprint. The reuse of newspaper waste not only supports environmental sustainability but also promotes economic opportunities by creating a new market for recycled materials.

## 2 Materials and methods

### 2.1 Materials

DAO enzyme, HPMC, sodium hydroxide (NaOH), sodium hypochlorite (NaOCl), TMB, HRP enzymes and acetic acid were received from Sigma-Aldrich. The enzyme (*Trichoderma reesei* ATCC26921) used in the study was obtained from Sigma Chemical Co., USA, with specific enzyme activity of around 700 U/g. Phosphate buffered saline (PBS) solution was received from AMRESCO-VWR. Analytical-grade chemicals and solvents were all utilized without additional purification. Kombucha tea leaf was obtained from a local market for making tea solution [vegain kombucha* 94.50 % (kombucha Culture*, black tea, green tea, organic cane sugar 4 %), sweet osmanthus 1 %, *bacillus coagulans* 0.5 %) *organically produced]. All experiments used deionized water obtained from a Milli-Q system.

### 2.2 Extraction and purification of cellulose fiber from newspaper waste

Initially, newspaper waste was cut into small pieces, soaked in deionized water for 24 h, and treated with a 10 *w*/w % NaOH solution (Newspaper wet weight: NaOH (1:5)) in a magnetic stirrer at 700 rpm at 80 °C for 3 h, partially adapting a method by Srasri et al. (Srasri et al., 2018). The dispersion was subject to vigorous mechanical stirring at 700 rpm. The resultant was placed in a filtration bag (100 μm) and thoroughly rinsed with deionized water for at least six times to remove any residual. After that, the dispersion was treated with deionized water: NaOCl solution: acetic acid (3:1:0.35) in a magnetic stirrer at 700 rpm at 85 °C for 3 h, partially adapting a method by Yıldız et al. (Dursun & Yıldız, 2023). Then, it was placed in a filtration bag (100 μm) and thoroughly rinsed with deionized water several times to remove excess chemicals and obtain bleached cellulose fiber. Then it was stored in the refrigerator. Fig. S1 depicts images of the whole process of extracting and purifying cellulose fiber from newspaper waste. All percentage solutions mentioned in this study are calculated based on weight (*w*/w).

### 2.3 Enzymatic hydrolysis of cellulose fibers from newspaper waste

The cellulosic paste, primarily composed of microfibrils, was further transformed into nanocrystals by treating it with commercially available cellulase. A quantity of 10 g (wet weight) of the paste was suspended in 200 mL of acetate buffer (0.1 M, pH 5.0), to which 1 mL of cellulase was added with gentle stirring. This suspension was maintained at 50 °C for 24 h in an incubator. Finally, the mixture was centrifuged at 10,000 rpm (4 °C for 16 min) to terminate the enzymatic reaction and collect the cellulosic fragments.

### 2.4 Enzyme-hydrolyzed cellulose immobilization of DAO

For DAO immobilization, 20 mg of the enzyme-hydrolyzed cellulose was suspended in 10 mL of 100 mM phosphate buffer (pH 7.0) containing 20 mg of purified DAO. The mixture was incubated at 4 °C for 3 h under gentle stirring (200 rpm), partially adapting a method by Verma et al. (Verma et al., 2020a). Subsequently, the DAO-immobilized cellulose was then lyophilized for 48 h in a freeze dryer. The resulting DAO-immobilized enzyme-hydrolyzed cellulose was stored at 4 °C until further use.

### 2.5 HPMC/DAO-immobilized enzyme-hydrolyzed cellulose film sensor formation

HPMC/enzyme-hydrolyzed cellulose suspensions of bases HPMC, enzyme-hydrolyzed cellulose was prepared using the casting technique, and DAO-immobilized enzyme-hydrolyzed cellulose suspensions of bases HPMC. 1.5 and 3 *w*/w % film-forming solutions were prepared separately by fixed enzyme-hydrolyzed cellulose and DAO-immobilized enzyme-hydrolyzed cellulose concentration at 0.1 w/w %, as well as a control for examining effects in the absence of enzyme-hydrolyzed cellulose and DAO (see Table 1). The procedure was conducted based on the guidelines of previous work reported by Kim et al. (Kim et al., 2008). HPMC was used as polysaccharide-based polymer and then added promptly with the enzyme-hydrolyzed cellulose. The film-forming solution was magnetic stirred at 750 rpm, RT for 6 h to ensure a homogeneous solution. Finally, the 1.5 and 3 *w*/w % of HPMC/Enzyme-hydrolyzed cellulose-base film was obtained by pouring 4 ml of each film-forming suspension into ten polystyrene Petri dishes (2 cm in diameter). All films were carefully cut into the circular shape of a diameter of 7 mm and placed in an air-conditioned laboratory at 25 °C and 75 % relative humidity for 72 h before the characterization test. Additionally, it should be noted that the illustration depicting the extraction and purification of cellulose fiber from newspaper waste, the enzymatic hydrolysis of these fibers, the enzyme-hydrolyzed cellulose immobilization of DAO, and the HPMC/DAO-immobilized enzyme-hydrolyzed cellulose film sensor formation is presented in the graphical abstract.

**Table 1:** HPMC/enzyme-hydrolyzed cellulose suspension solution conditions prepared for film formation.

Formula	HPMC (%, *w*/*v*)	Enzyme-hydrolyzed cellulose (*w*/w %)	DAO-immobilized enzyme-hydrolyzed cellulose (w/w %)
Control	3	–	–
HPMC−1.5	1.5	0.1	–
HPMC-3	3	0.1	–
HPMC/DAO-1.5	1.5	–	0.1
HPMC/DAO-3	3	–	0.1

## 3 Characterization

### 3.1 Characterization technique

Transmission electron microscopy (TEM) micrographs of the film samples were taken with a JEOL 100CX-2 transmission electron microscope at an accelerating voltage of 100 kV. Samples were diluted and prepared by dropping the sample suspension on a carbon-coated grid and allowing it to dry. The morphology was monitored by scanning electron microscope (SEM, Quanta 250 microscope, Japan). The specimen was coated with gold using a sputtering device (JEOL, JFC 1200, Japan) prior to the SEM observation. A magnification of 1 K and 10 K was used. The surface morphology of the cotton thread working electrode were characterized using a JSM-IT300HR scanning electrode microscope (Japan Electron Optics Laboratory Co., Ltd., Japan) and a LEXT OLS5000 laser scanning confocal microscope (Olympus Corporation, Japan). The crystallographic structure was analyzed an X-ray diffractometer (AXS model D8 Advance, Bruker, Germany). The operating conditions employed nicked-filtered CuKα radiation, and X-ray diffraction profiles were obtained for 2θ ranging from 5° to 80°, with a scanning rate of 0.02/min and time/step of 0.2 s. The sample were stored in a desiccator to prevent moisture absorption. The thermal behavior was investigated using DSC (NETZSCH DSC 204 F1 Phoenix, Germany). The sample was placed in aluminum pans and purged with nitrogen gas at a flow rate of 40 mL·min^−1^. The temperature was set to 30 °C to 250 °C, with a flow rate of 10 °C·min^−1^. The data was reported as glass transition temperature, melting temperature, and specific heat capacity. Thermogravimetric analysis (TGA) was performed using TGA 2, Mettler Toledo, Switzerland, under flowing nitrogen atmosphere at flow rate of 20 ml/min. The nitrogen was used to avoid contamination and condensation on the samples. Approximately 15 mg of sample was heated from 40° to 600 °C at a heating rate of 10 °C/min. The sample weight loss and weight loss rate were continually monitored in relation to temperature. Only until an isothermal state had been achieved was the analysis conducted. The swelling characteristics of the samples were investigated using a gravimetric technique. Film samples with a total surface area of 2 cm^2^ were entirely immersed in deionized water for 30 s to 30 min. The swelling time over a period of 30 min was carried out by immersing the film in deionized water to investigate the stability of films. Following the deionized water contact time, the excess deionized water from the swollen samples was carefully wiped out with clean tissue paper before being weighted using an analytical scale. According to Eq. (1), the swelling degree is determined by the mass change and data were reported as statistical average and standard deviation.

(1) $$\text{Swelling ratio}\;\left(\%\right)=\left[\left(W_{w}–W_{d}\right)/W_{d}\right]\;x\;100$$

where W_w_ is the weight of the swollen cellulose-base hydrogel at submersion time and W_d_ is the initial weight of the dry cellulose-base hydrogel.

### 3.2 Histamine sensor experiment

The histamine concentration analysis of the reference standard and kombucha tea solution was performed using indirect quantification methods via UV-VIS spectroscopy. The analysis utilized a PEAK Instruments C-7100 UV/VIS spectrophotometer, configured with a slit width of 2.0 nm and a light source set at 340.8 nm, covering a wavelength range from 190 to 1100 nm. This study employed histamine dihydrochloride as a histamine reference, which was selected based on its established pharmacological properties and its ability to mimic endogenous histamine responses in food products (Altafini et al., 2022). A standard curve for histamine dihydrochloride was established using UV-VIS spectroscopy to ensure the quality of the quantification. A stock solution of 5.00 mg/mL was prepared by dissolving 500.0 mg of histamine dihydrochloride in 100 mL of deionized water. Standard solutions with concentrations of 1.00, 2.00, 3.00, 4.00, and 5.00 mg/mL were created through serial dilution. Absorbance measurements were taken at 210 nm, using phosphate buffer as a blank. All measurements were conducted in triplicate with quartz cuvettes of 1 cm path length. A standard curve was constructed by plotting the mean absorbance against the corresponding concentrations. Linear regression analysis was performed to derive the equation of the line. The method was validated with quality control samples, and the determined linear range of 1, 2, 3, 4, and 5 mg/mL was determined (see Fig. S2). This approach allows for the quantification of histamine dihydrochloride in unknown samples, facilitating the determination of histamine concentration in kombucha tea solutions. For the experimental setup of histamine sensing in kombucha, a cellulose-based sensor was employed. HRP with an activity of 297 units/mg was prepared as a stock solution of 100 units in 5 mL, then diluted 100-fold to achieve a working concentration of 1 unit/mL in 10 mL of PBS buffer. Solutions of TMB were prepared at a concentration of 1 mM. The histamine detection was conducted using a colorimetric enzyme-linked assay. The assay protocol involved adding 100 μL of the kombucha tea sample, followed by 100 μL each of HRP (1 U/mL) and TMB (1 mM). This reaction cascade induced a color change directly correlating with the histamine concentration in the kombucha sample. The reaction time was evaluated as an immediate change, with a stable color observed within 30 s. KEPLER (KCDM-100) device was used to quantify the visible color transition of the film. Results were reported in terms of L* (lightness), a* (redness), and b* (yellowness), following the CIELAB methodology. Color readings were performed in triplicate under identical lighting conditions, and the average values were recorded. The instrument was standardized using a standard white tile (L*92.93, a*-0.92, b*1.48). The overall color difference (ΔE* value) was calculated using the eq. (2):

(2) $${\mathrm{\Delta E}}^{*}={\left[{\left({\mathrm{\Delta L}}^{*}\right)}^{2}+{\left({\mathrm{\Delta a}}^{*}\right)}^{2}+{\left({\mathrm{\Delta b}}^{*}\right)}^{2}\right]}^{0.5}$$

where ΔL*, Δa*, and Δb* are the differences in the corresponding color parameters between the film samples and the standard white tile. Each film sample was measured in triplicate.

### 3.3 Statistical analysis

Statistical analysis was performed with the Statistical Package for the Social Sciences (SPSS for Windows version 16.0, SPSS Inc., Chicago, IL, USA). Data was analyzed using one-way ANOVA and Duncan's multiple range tests to identify mean differences. Post-hoc tests were adjusted to a significance threshold of 0.05 (*P* ≤ 0.05) for multiple comparisons.

## 4 Results and discussion

### 4.1 Physicochemical characteristics of enzyme-hydrolyzed cellulose and HPMC/enzyme-hydrolyzed cellulose-base films

**Fig. 1:**
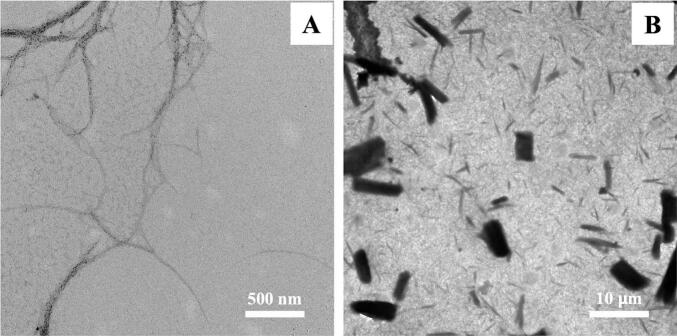
TEM images of enzyme-hydrolyzed cellulose (A) and non-hydrolyzed cellulose (B).

The abundant thick bundles of cellulose fibers of the non-hydrolyzed cellulose (B), which corresponded to the TEM images are showed in Fig. 1. In contrast with enzyme-hydrolyzed cellulose (A) that show an increase of single fibers. The integrated structure of cellulose fiber bundles was broken into separated fibers and many small fiber bundles by penetrating of hydrogen ions (H^+^) into amorphous cellulose molecules promoting cleavage of covalent or glycosidic linkage/bonds, therefore releasing single crystallites.

**Fig. 2:**
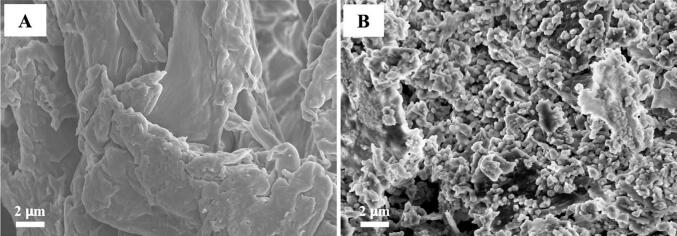
SEM images of enzyme-hydrolyzed cellulose pre-immobilization of DAO (A) and enzyme-hydrolyzed cellulose pre-immobilization of DAO (B). Bar size for SEM images; (A) 2 nm and (B) 2 μm.

To elucidate the surface characteristics of cellulose fibers before and after enzyme attachment, we conducted a comparative analysis using scanning electron microscopy (SEM). Two representative micrographs were captured to highlight the differences in surface features. The first micrograph (Fig. 2A) illustrates the topography of unmodified cellulose fibers. These fibers exhibit a predominantly smooth surface with subtle, inherent irregularities typical of cellulosic structures. The elongated fibrous formations display a generally uniform texture without any notable particulate features at the magnification used. In contrast, the second micrograph (Fig. 2B) reveals the cellulose fibers post-immobilization of DAO. This image clearly shows the presence of numerous spheroidal structures adorning the fiber surfaces (Verma et al., 2020b). These globular entities, identified as immobilized DAO molecules, manifest as distinct particles affixed to the underlying cellulose substrate. The enzymatic spheroids, with diameters in the range of X to Y nanometers, are discernibly separate from the base fiber structure, although their distribution across the surface is not entirely homogeneous. The stark contrast between these two micrographs clearly illustrates the successful immobilization of DAO on the cellulose fibers, demonstrating significant morphological changes that indicate effective binding and integration of the enzyme within the fiber matrix. The preservation of the enzyme's globular morphology suggests that the immobilization process likely maintained the protein's structural integrity, which is essential for its catalytic function. This SEM-based morphological assessment not only confirms the efficacy of our immobilization protocol but also offers valuable insights into the surface properties of the enzyme-modified cellulose fibers. Physical immobilization methods for diamine oxidase (DAO) on cellulose fibers utilize non-covalent interactions to anchor enzymes, often preserving their catalytic activity (Liu & Chen, 2016). DAO immobilization via adsorption represents a facile approach among physical immobilization techniques. Adsorption onto solid supports is notably simpler to execute compared to other immobilization methods (Orelma et al., 2012). Unlike entrapment, adsorption typically involves the initial synthesis of cellulose matrices followed by enzyme immobilization through adsorption onto these matrices. This technique leverages physical interactions between the matrix and enzyme, including van der Waals forces, ionic interactions, and hydrogen bonding (Hernandez & Fernandez-Lafuente, 2011). A key advantage of adsorption is its tendency to preserve the native structure of enzymes, minimizing perturbations to their active sites and thereby maintaining catalytic activity [87]. This non-disruptive nature of adsorption contributes to its widespread application in enzyme immobilization, particularly for DAO, where retention of enzymatic function is crucial.

**Fig. 3:**
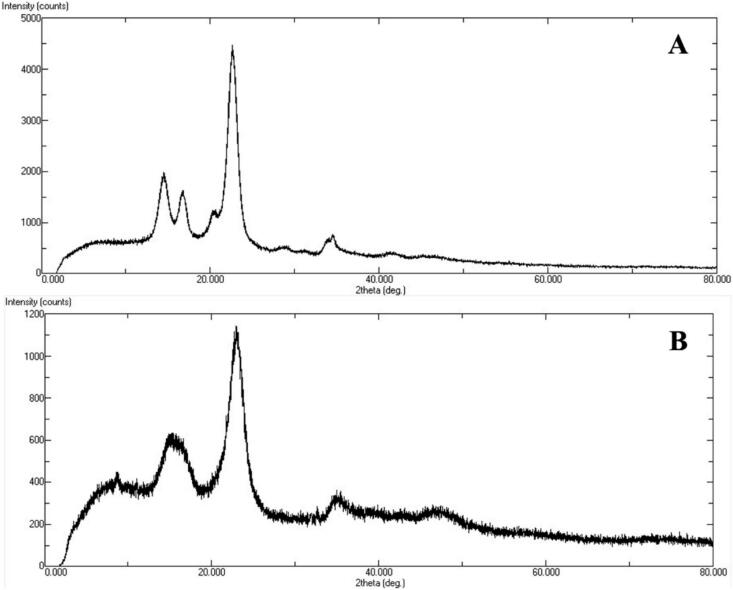
XRD patterns of non-hydrolyzed cellulose (A) and enzyme-hydrolyzed cellulose (B).

The XRD analysis of the solid state of non-hydrolyzed cellulose (A) and enzyme-hydrolyzed cellulose (B) is shown in Fig. 3. The non-hydrolyzed cellulose has a crystalline structure, shown in Fig. 3 (A), as indicated by a pattern of sharp diffraction peaks between 10 and 40 at 2θ degree (Islam et al., 2011). The enzyme-hydrolyzed cellulose showed higher amorphous patterns in their XRD patterns, as illustrated in Fig. 3 (B), while still exhibiting modestly sharp diffraction peaks between 10 and 40 at 2θ degree.

**Fig. 4:**
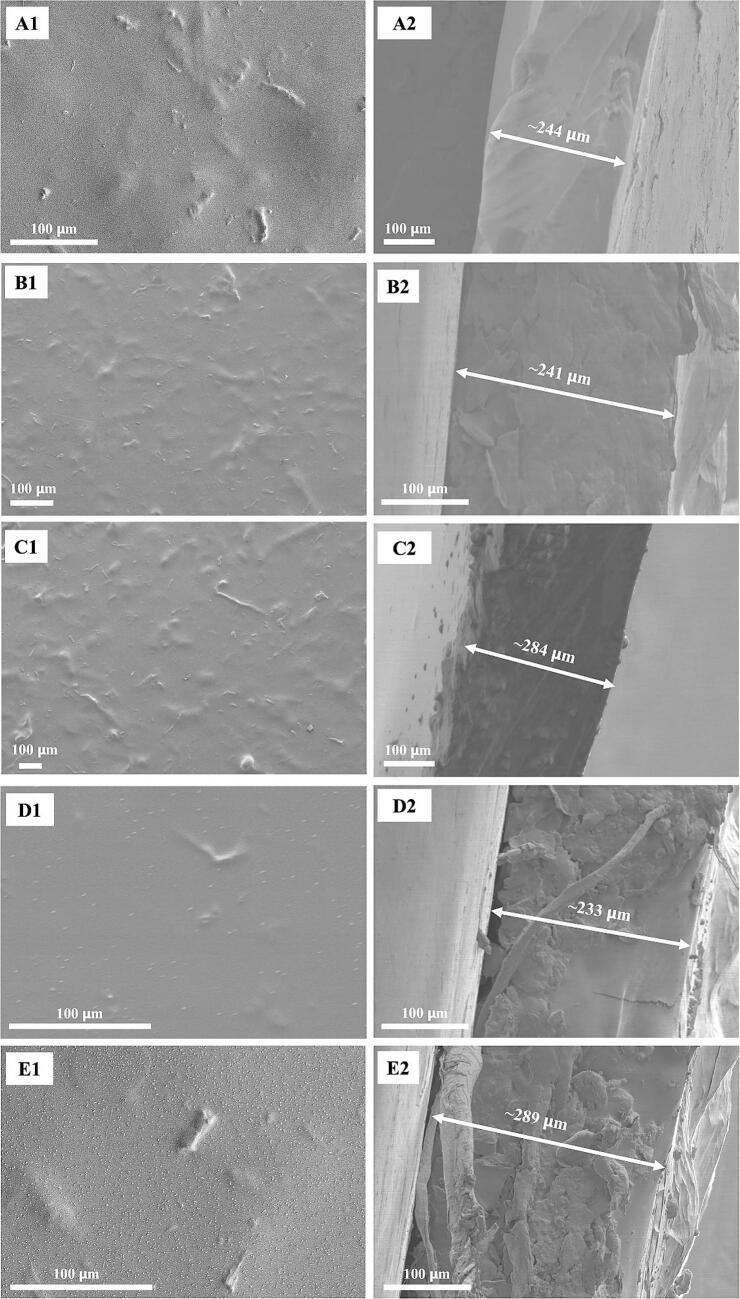
SEM images of HPMC/enzyme-hydrolyzed cellulose-base film sensor; (A1) control, (B1) HPMC-1.5, (C1) HPMC-3, (D1) HPMC/DAO-1.5, and (E1) HPMC/DAO-3; SEM cross-section images of (A2) control, (B2) HPMC-1.5, (C2) HPMC-3, (D2) HPMC/DAO-1.5, and (E2) HPMC/DAO-3.

The microstructure of HPMC/enzyme-hydrolyzed cellulose-base film sensor is shown in Fig. 4. Each film was presented with a similar morphology. It was described as a thin layer film with homogeneous enzyme-hydrolyzed cellulose particles dispersed at cross sections throughout the layer (see Fig. 4 B2, C2, D2, and E2), while the control film is the absence of cellulose fiber. 1.5 and 3 *w*/w % HPMC/enzyme-hydrolyzed cellulose-base films and 1.5 and 3 w/w % HPMC/DAO enzyme-hydrolyzed cellulose-base films each have slightly different film thicknesses. The 1.5 and 3 w/w % DAO-immobilized enzyme-hydrolyzed cellulose films showed a small circular shape of homogeneously dispersed DAO particles at the film surface (see Fig. 4 D2 and E2). Film thicknesses were approximately 233 and 289 μm. The 3 *w*/w % film showed a higher thickness compared to the 1 *w*/w % film. Therefore, the presence of enzyme-hydrolyzed cellulose resulted in a clearly visible spot of particles dispersed in the in the microstructure of the cellulose-base film.

**Fig. 5:**
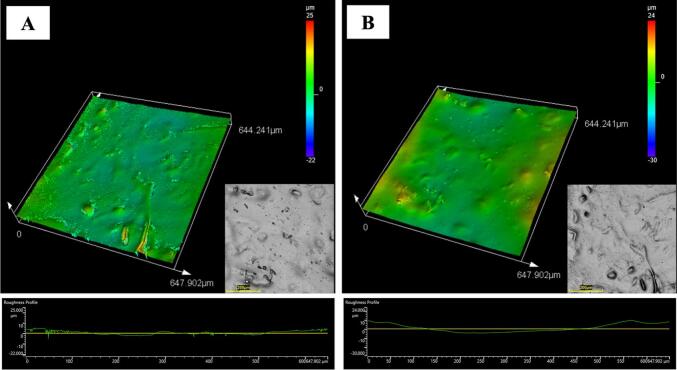
Three-dimensional confocal microscope images of 1.5 w/w % HPMC/DAO-immobilized enzyme-hydrolyzed cellulose film and 3 w/w % HPMC/DAO-immobilized enzyme-hydrolyzed cellulose film. The x-y-z dimensions of images are 647.902 μm × 644.24 μm × 25 μm.

From now on, we will discuss the characterization of only the HPMC/DAO-immobilized enzyme-hydrolyzed cellulose films in order to focus precisely on how DAO functions on the film appearance and their properties. The surface roughness of HPMC/DAO-immobilized enzyme-hydrolyzed cellulose films with 1.5 *w*/w % and 3 w/w % HPMC concentrations was analyzed using laser scanning confocal microscopy, as illustrated in Fig. 5. The average surface roughness values for the 1.5 *w*/w % and 3 w/w % HPMC films were determined to be 1.71 ± 1.20 and 2.54 ± 0.91, respectively. Notably, the 1.5 w/w % HPMC film exhibited more consistent surface roughness compared to its 3 w/w % counterpart. This enhanced consistency can be attributed to the reduced thickness of the 1.5 w/w % film, which likely promotes a more uniform distribution of the HPMC/DAO-immobilized enzyme-hydrolyzed cellulose components during film formation.

### 4.2 Thermal property of enzyme-hydrolyzed cellulose and HPMC/DAO-immobilized enzyme-hydrolyzed cellulose film

**Fig. 6:**
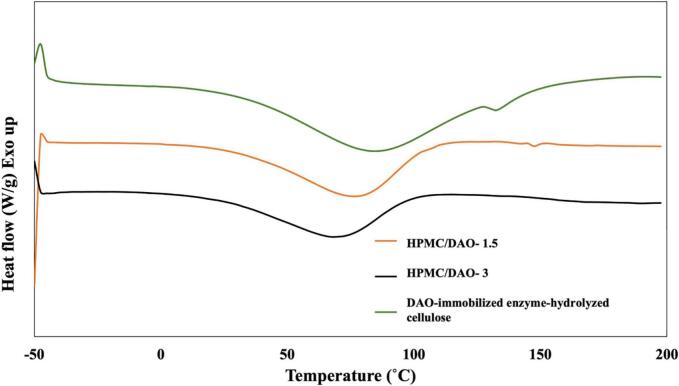
DSC thermogram of 1.5 *w*/w % HPMC/DAO-immobilized enzyme-hydrolyzed cellulose film, 3 *w*/w % HPMC/DAO-immobilized enzyme-hydrolyzed cellulose film, and DAO-immobilized enzyme-hydrolyzed cellulose.

In order to predict the thermal behavior of the enzyme-hydrolyzed cellulose and the enzyme-hydrolyzed cellulose loaded into HPMC-based film, DSC technique was employed. Fig. 6 presents the differential scanning calorimetry of enzyme-hydrolyzed cellulose loaded into HPMC-based film. It was found that all of characteristic peaks were presented in the similar form. No significant change of peak regarded to the presence of curcumin was observed. This was probably due to the small amount. It may adhere to hydroxyl group of HPMC by hydrogen bonding throughout hydrogel network. The crystallization temperature (T_c_) of 1.5 *w*/w % HPMC/DAO-immobilized enzyme-hydrolyzed cellulose film, 3 w/w % HPMC/DAO-immobilized enzyme-hydrolyzed cellulose film, and DAO-immobilized enzyme-hydrolyzed cellulose were measured at 76.45 °C, 68.62 °C, and 83.45 °C, respectively. These temperatures indicate the points at which the respective materials undergo a transition from an amorphous state to a crystalline solid, reflecting variations in the molecular interactions and structural organization within the films. This crystallization process is exothermic and results in a distinct peak in the DSC signal, highlighting the thermal stability and phase transitions of the materials studied. The results reveal an endothermic peak (68–83 °C) for all samples, where cellulose, in agreement with another study (Veeramachineni et al., 2016). The enthalpy for this process relates to evaporation of bound water to the polysaccharide.

**Fig. 7:**
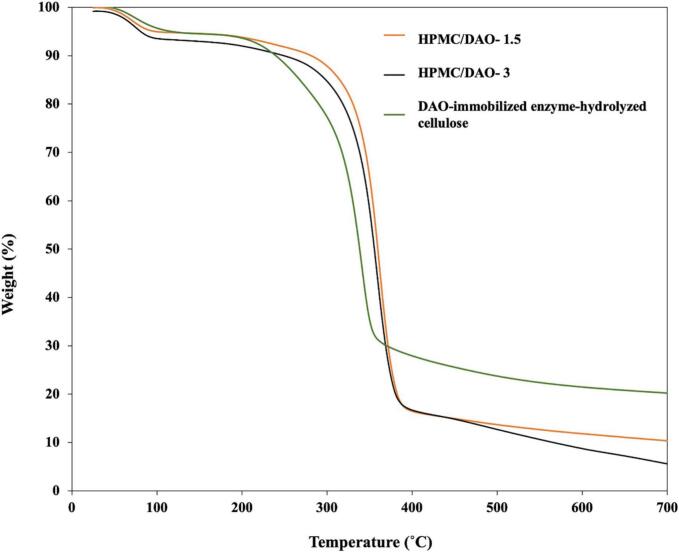
TGA curves of 1.5 w/w % HPMC/DAO-immobilized enzyme-hydrolyzed cellulose film and 3 w/w % HPMC/DAO-immobilized enzyme-hydrolyzed cellulose film, and DAO-immobilized enzyme-hydrolyzed cellulose.

The TGA technique was employed to conduct a thermal analysis in terms of the thermal degradation state of the enzyme-hydrolyzed cellulose and the DAO-immobilized enzyme-hydrolyzed cellulose loaded into HPMC-based film. The TGA curves are shown in Fig. 7. In Fig. 5, the mid-point temperatures that represent the decomposed temperatures of 1.5 *w*/w % HPMC/enzyme-hydrolyzed cellulose-base film, 3 w/w % HPMC/DAO-immobilized enzyme-hydrolyzed cellulose film, and DAO-immobilized enzyme-hydrolyzed cellulose are 357.05 °C, 358.08 °C, and 333.79 °C, respectively.

### 4.3 Swelling and water retention of HPMC/enzyme-hydrolyzed cellulose-base films

**Fig. 8:**
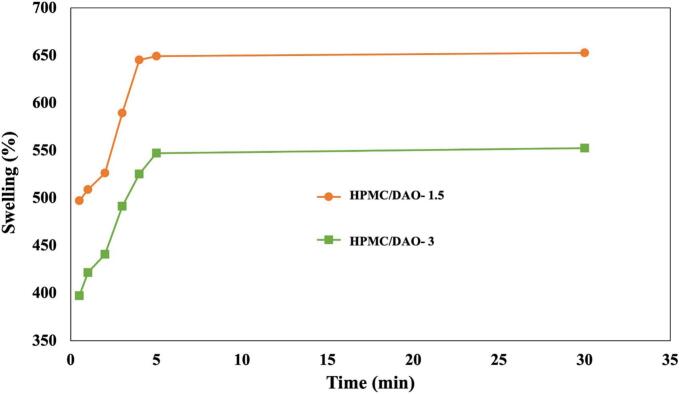
Swelling behavior of 1.5 w/w % HPMC/DAO-immobilized enzyme-hydrolyzed cellulose film, and 3 w/w % HPMC/DAO-immobilized enzyme-hydrolyzed cellulose film.

To investigate the feasibility of HPMC/DAO-immobilized enzyme-hydrolyzed cellulose film as a platform of sensing material, swelling behavior experiment was conducted from 30 s to 30 min. Fig. 8 reports the behavour during the swelling process of 1.5 and 3 *w*/w % HPMC/DAO-immobilized enzyme-hydrolyzed cellulose film and 3 w/w % HPMC/DAO-immobilized enzyme-hydrolyzed cellulose film within 30 min. It was observed that the swelling behavior in deionized water can be classified into two regions. At initial stage, the swelling behavior was very rapid within 5 min. This was probably due to the presence of abundant of hydroxyl group of HPMC. It can create the H-bond when the film was immersed into deionized water. After that, the plateau region was observed from 5 to 30 min. It therefore exhibited excellent dimensional stability. Furthermore, for 1.5 w/w % HPMC/DAO-immobilized enzyme-hydrolyzed cellulose film, the level of swelling characteristic was slightly shifted to higher. Because of the slight thin layer of film thickness, it may create the H-bond formation along with the pendent group of HPMC-base film. The insertion of this small molecule significantly enhances the hydrogel's properties, leading to a pronounced initial swelling behavior that eventually stabilizes, indicating a robust interaction with the hydrogel matrix. Upon immersion in deionized water, the molecule dissociates into mobile ions, which facilitates its removal from the hydrogel network. This critical dissociation not only alters the internal dynamics of the hydrogel but also results in superior swelling characteristics, optimizing the interaction between the remaining components and the surrounding medium.

### 4.4 Histamine sensor experiment

**Fig. 9:**
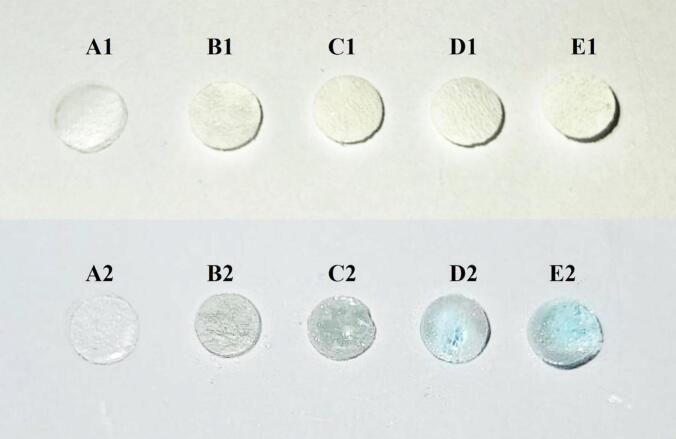
Visual appearance of HPMC/enzyme-hydrolyzed cellulose-base film sensor before histamine sensing of kombucha tea; (A1) control, (B1) HPMC-1.5, (C1) HPMC-3, (D1) HPMC/DAO-1.5, and (E1) HPMC/DAO-3; and after; (A2) control, (B2) HPMC-1.5, (C2) HPMC-3, (D2) HPMC/DAO-1.5, and (E2) HPMC/DAO-3.

Histamine sensor experiment of the HPMC/enzyme-hydrolyzed cellulose-base film as shown in Fig. 9. Histamine detection in kombucha tea samples was performed using a colorimetric enzyme-linked assay. The method involved reacting histamine with DAO to produce hydrogen peroxide (H_2_O_2_). The resulting H_2_O_2_ then reacted with HRP, which catalyzed the oxidation of the colorless substrate TMB using H_2_O_2_ as the oxidizing agent (Rentzos et al., 2024). This oxidation reaction led to color development proportional to the original histamine concentration in the sample. The intensity of the color produced directly correlated with the histamine concentration, allowing for sensitive colorimetric detection. In this assay, peroxidase acted as a catalyst for the oxidation of the substrate, while the H_2_O_2_ produced from the initial histamine reaction served as the oxidizing agent. The resulting color development from the oxidized substrate provided a quantitative indication of the original histamine content in the kombucha tea sample. The concentration of histamine in the kombucha tea sample as an amount of 100 μL of each replication was calculated by using the correlation plot of the histamine standard UV-VIS spectroscopy technique. The concentration of the kombucha tea sample, 3.30 mg/mL, was calculated from the standard curve plot of histamine dihydrochloride reference, according to the method described in Section 3.2. The results show that the DAO-immobilized enzyme-hydrolyzed cellulose-base film sensor (Figs. 9 D2 and E2) was color changed and turned its color from opaque white to translucent blue, especially in the HPMC/DAO-immobilized enzyme-hydrolyzed cellulose-base film sensor at 3 *w*/w % (HPMC/DAO-3) (Fig. 9 E2). Because the higher the concentration of suspension, the higher the amount of DAO presence could affect the reaction of histamine as an analyzer.

During the enzymatic reaction, in the presence of peroxidase and H_2_O_2_, the HPMC/DAO-immobilized enzyme-hydrolyzed cellulose-base film sensor gets oxidized, and a blue spot is obtained. Histamine level were measured in kombucha tea with favorable results (relative to the control). The study concludes by underlining the environmental sustainability of the HPMC-based hybrid material and its pivotal role in ensuring food safety through accurate histamine detection in kombucha. The visible color shift of the film was quantified instrumentally utilizing KEPLER (KCDM-100) to accomplish quantitative analysis for histamine sensing while avoiding subjectivity from naked eye examination. In the CIELAB color system, parameter b* measures the blue color negative and yellow color positive values, which are more closely related to the actual color shift of the studied sample than parameter a*, which measures the red color positive and green color negative values (Carpenter et al., 2017). The histamine sensing process of the films was strongly related to the surface reactions and was well characterized by the color evolution of the film surface. The histamine sensing material, based on the reaction of histamine, DAO-immobilized enzyme-hydrolyzed cellulose, HRP, and the TMB colorless leuco dye substrate solutions, was generated by the change of film color. The change in the film color of the HPMC/DAO-1.5 and HPMC/DAO-3 resulted from a significant contribution from the b* values and the ΔE* value as shown in Table 2.

**Table 2:** b* parameter and ΔE* value obtained from CIELAB data of the HPMC/enzyme-hydrolyzed cellulose-base film sensor upon sensing to kombucha tea in cooperation with HRP and TMB (*n* = 3).

Film sample	b*	ΔE* value
Control	-1 ± 0.05^c^	80.34
HPMC-1.5	2.75 ± 0.03^f^	67.43
HPMC-13	1.70 ± 0.01^d^	67.85
HPMC/DAO-1.5	−6.91 ± 0.02^b^	79.41
HPMC/DAO-3	−8.85 ± 0.01^a^	80.21

Note: All values are given as mean ± SD, n = 3 for each group. Different superscript letters within the same row are significantly difference (*P* < 0.05).

In comparing the response of the b* value with film samples, the histamine sensitivity of the film was determined. The b* value decreased dramatically with a significant difference (*P* < 0.05) when exposed to a kombucha tea sample of 100 μL, which is the color conversion from opaque white to transparent blue. As the concentration of DAO-immobilized enzyme-cellulose was increased in the films of HPMC/DAO-1.5 and HPMC/DAO-3, the intensity of the blue color gradually increased, as shown in Fig. 9, corresponding to a radical decrease in the b* parameter (see Table 2). and it is clearly seen in Table 2 that the b* values of the HPMC/DAO-3 film showed the highest sensitivity and a significant decrease in sensitivity with the kombucha tea sample. Since the film color was completely changed, the ΔE* value can confirm the color difference in comparison between those of film samples. The film color with the cooperated DAO-immobilized enzyme-cellulose at 3 % *w*/w % changed and exhibited the lowest b* value (−8.85 ± 0.01); this indicated that the minimum b* value that can be displayed in blue. The b* value decreased dramatically with a significant difference (*P* < 0.05) when exposed to a kombucha tea sample of 100 μL. This resulted in a color conversion from opaque white to transparent blue.

TMB, one of the most common chromogenic substrates used in peroxidase-based assays, is itself colorless. Upon oxidation by peroxidase in the presence of hydrogen peroxide (H_2_O_2_), TMB undergoes a reaction that results in the formation of a blue-colored oxidation product (Bai et al., 2020; Moon et al., 2020; Wang et al., 2018). The reaction involves the one-electron oxidation of TMB by complexed H_2_O_2_/peroxidase to form an aromatic cation radical intermediate. This electrically charged intermediate is resonance stabilized, giving it its characteristic blue color (Josephy et al., 1982). Specifically, the reaction can be summarized as in eq. 3:

(3) $$\mathrm{TMB}+H_{2}O_{2}\to {\mathrm{TMB}}^{\cdot +}+H_{2}O$$

where TMB^·+^ represents the resonance stabilized cation radical product. Through the loss of a second electron, the unstable cation radical undergoes further oxidation to form the more stable dication as in eq. 4:

(4) $${\mathrm{TMB}}^{\cdot +}\to {\mathrm{TMB}}^{2+}$$

It is this blue-colored dication that accumulates over time, producing the visible color change that allows for spectrophotometric or visual detection of the analyte using this chromogenic system (Mitran et al., 2020). The use of TMB as a peroxidase substrate thus exploits its ability to serve as an electron donor in the oxidation reaction, becoming colored only after enzymatic transformation catalyzed by H_2_O_2_. This reaction principle underlies its widespread application as a sensitive indicator in countless bioanalytical and diagnostic assays. The developed enzyme-hydrolyzed cellulose-based sensor technology has significant potential for application in various food matrices beyond kombucha tea. For instance, it could be effectively utilized for histamine detection in seafood, where histamine levels are critical for food safety. Additionally, the sensor may be adapted for use in dairy products and other fermented foods, where similar concerns regarding histamine content exist. By broadening the scope of application, this technology could enhance food safety measures across multiple sectors and contribute to consumer health.

## 5 Conclusion

This study highlights a significant advancement in histamine sensing technology through the development of a cellulose-based material derived from newspaper waste for detecting histamine in kombucha. The hybrid material, combining polysaccharides and cellulose from newspaper waste, was utilized to create an effective sensor. The HPMC/enzyme-hydrolyzed cellulose-based film sensor demonstrated reliable colorimetric detection of histamine in kombucha samples, with the DAO-immobilized sensors at a 3 *w*/w % concentration (HPMC/DAO-3) exhibiting the most pronounced color change. The colorimetric enzyme-linked assay, which involved the DAO-H_2_O_2_-HRP-TMB reaction cascade, indicated the presence of histamine through color change. Increased suspension concentration used in film fabrication resulted in higher DAO levels, directly correlating color intensity to the fixed histamine concentration.

This study highlights the potential of these film sensors as effective, user-friendly, and cost-efficient tools for histamine detection in fermented beverages, paving the way for promising applications in food safety screening.

## CRediT authorship contribution statement

**Pongpat Sukhavattanakul:** Investigation, Formal analysis, Data curation, Conceptualization. **Nadnudda Rodthongkum:** Resources, Investigation, Formal analysis. **Sarute Ummartyotin:** Writing – review & editing, Writing – original draft, Supervision, Resources, Funding acquisition.

## Declaration of competing interest

The authors declare that they have no known competing financial interests or personal relationships that could have appeared to influence the work reported in this paper.

## Data Availability

Data will be made available on request.

## Appendix A Supplementary data

Supplementary material Image 1
