# Supplementary material for: Hybrid material of sustainable newspaper waste-derived cellulose for histamine sensing in kombucha tea
Source: Food Chem X. 2025 May 26;28:102578. doi: 10.1016/j.fochx.2025.102578 (PMC12159934; doi:10.1016/j.fochx.2025.102578)
Supplement: Supplementary file 1 — Supplementary material [file mmc1.docx]

**Supplementary materials**

**Hybrid material of sustainable newspaper waste-derived cellulose for**

**histamine sensing in kombucha tea**

Pongpat Sukhavattanakul ^a(*)^, Nadnudda Rodthongkum ^b, c^, Sarute Ummartyotin ^a, d(*)^

^a^ Department of Materials and Textile Technology, Faculty of Science and Technology, Thammasat University, Pathumtani, Thailand

^b^ Metallurgy and Materials Science Research Institute, Chulalongkorn University, Bangkok, Thailand

^c^ Center of Excellence in Responsive Wearable Materials, Chulalongkorn University, Soi Chula 12, Phayathai Road, Pathumwan, Bangkok, Thailand

^d^ Center of Excellence on Petrochemical and Materials Technology, Chulalongkorn University, Bangkok, Thailand


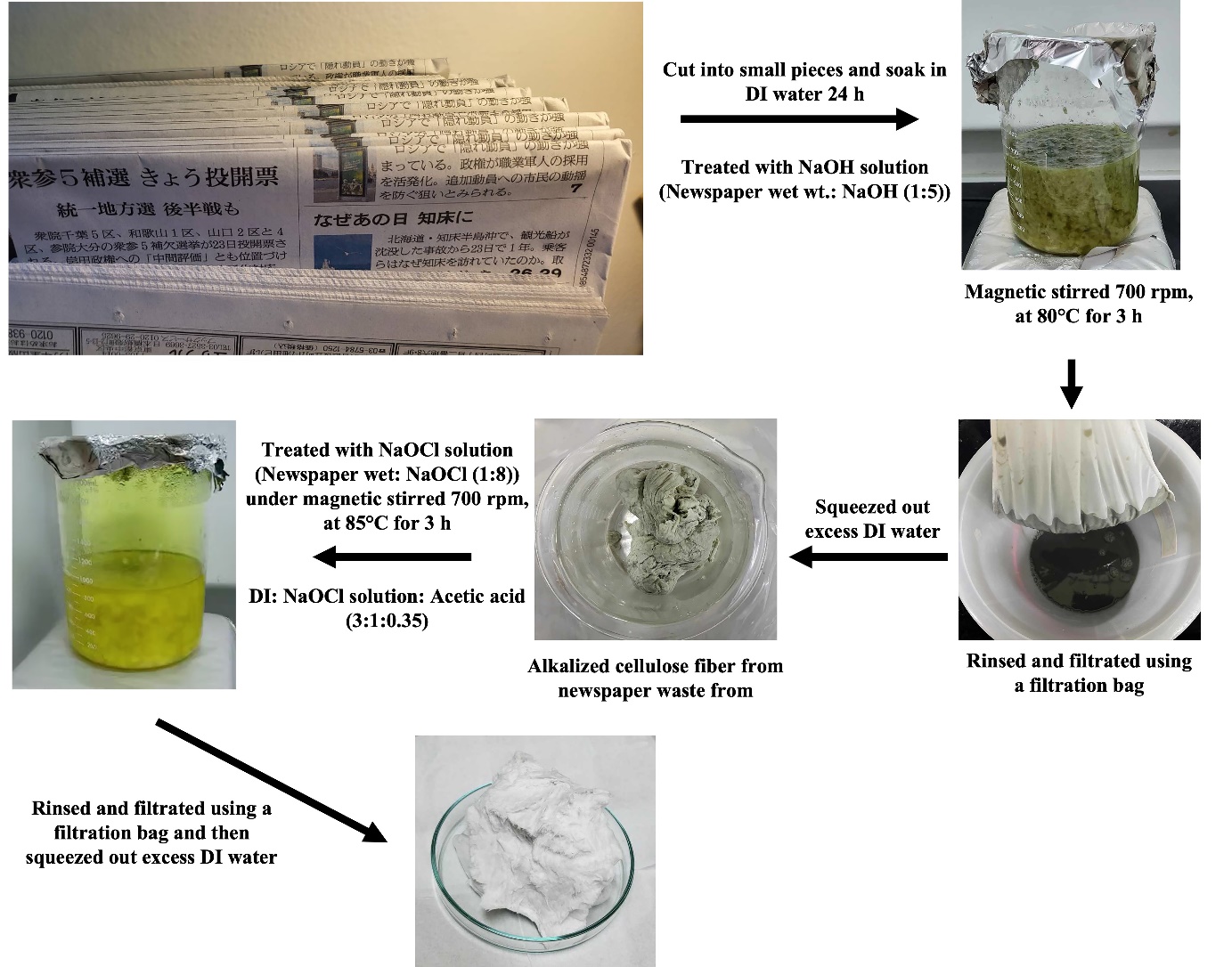


**Figure S1.** Photographs of the whole process of extracting and purifying cellulose fiber from newspaper waste.

**
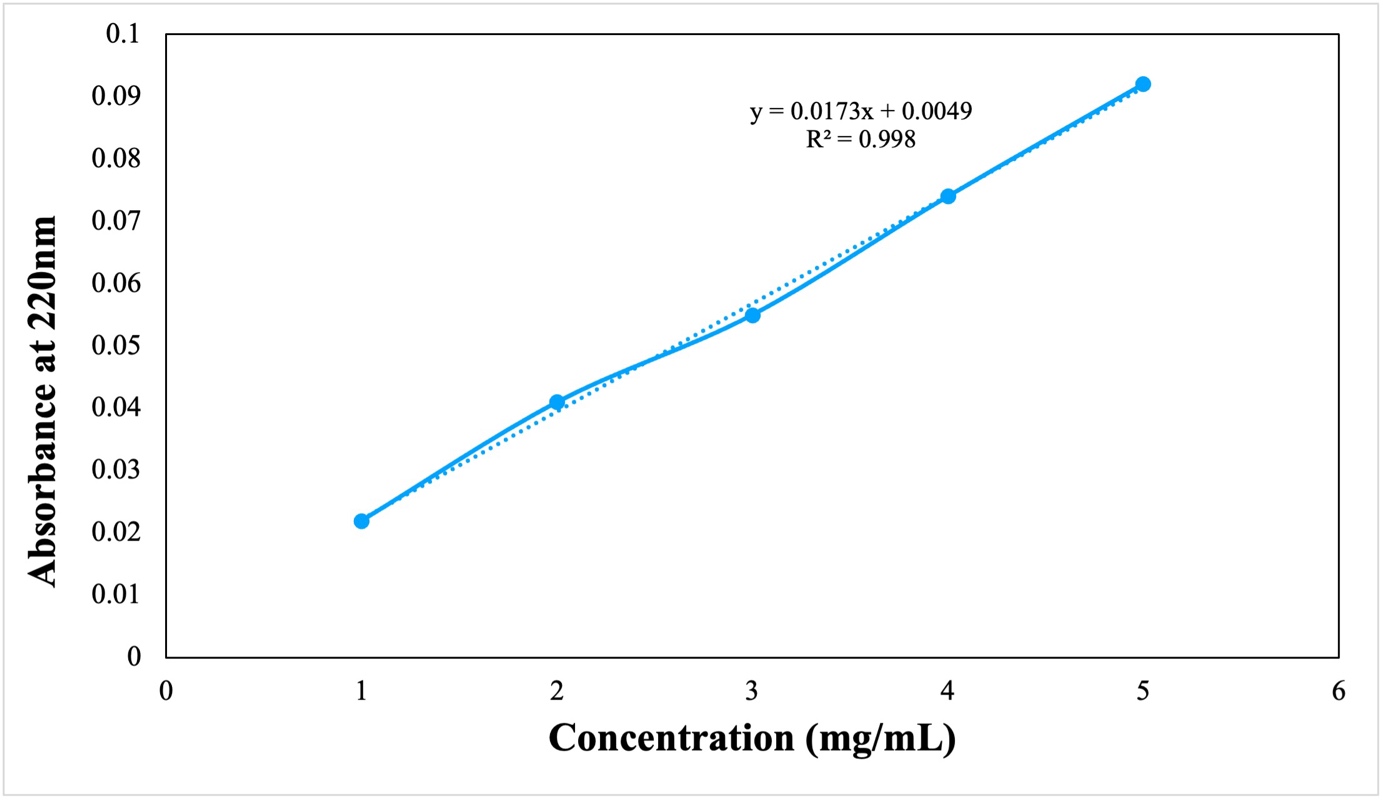
**

**Figure S2**. Standard curve for histamine dihydrochloride via UV-VIS spectroscopy, showing absorbance at 220 nm versus concentration (mg/mL).
